# Barnase as a New Therapeutic Agent Triggering Apoptosis in Human Cancer Cells

**DOI:** 10.1371/journal.pone.0002434

**Published:** 2008-06-18

**Authors:** Evelina Edelweiss, Taras G. Balandin, Julia L. Ivanova, Gennady V. Lutsenko, Olga G. Leonova, Vladimir I. Popenko, Alexander M. Sapozhnikov, Sergey M. Deyev

**Affiliations:** 1 Shemyakin and Ovchinnikov Institute of Bioorganic Chemistry, Russian Academy of Sciences, Moscow, Russia; 2 Engelhardt Institute of Molecular Biology, Russian Academy of Sciences, Moscow, Russia; 3 Institute of Gene Biology, Russian Academy of Sciences, Moscow, Russia; Weizmann Institute of Science, Israel

## Abstract

**Background:**

RNases are currently studied as non-mutagenic alternatives to the harmful DNA-damaging anticancer drugs commonly used in clinical practice. Many mammalian RNases are not potent toxins due to the strong inhibition by ribonuclease inhibitor (RI) presented in the cytoplasm of mammalian cells.

**Methodology/Principal Findings:**

In search of new effective anticancer RNases we studied the effects of barnase, a ribonuclease from *Bacillus amyloliquefaciens*, on human cancer cells. We found that barnase is resistant to RI. In MTT cell viability assay, barnase was cytotoxic to human carcinoma cell lines with half-inhibitory concentrations (IC_50_) ranging from 0.2 to 13 µM and to leukemia cell lines with IC_50_ values ranging from 2.4 to 82 µM. Also, we characterized the cytotoxic effects of barnase-based immunoRNase scFv 4D5-dibarnase, which consists of two barnase molecules serially fused to the single-chain variable fragment (scFv) of humanized antibody 4D5 that recognizes the extracellular domain of cancer marker HER2. The scFv 4D5-dibarnase specifically bound to HER2-positive cells and was internalized via receptor-mediated endocytosis. The intracellular localization of internalized scFv 4D5-dibarnase was determined by electronic microscopy. The cytotoxic effect of scFv 4D5-dibarnase on HER2-positive human ovarian carcinoma SKOV-3 cells (IC_50_ = 1.8 nM) was three orders of magnitude greater than that of barnase alone. Both barnase and scFv 4D5-dibarnase induced apoptosis in SKOV-3 cells accompanied by internucleosomal chromatin fragmentation, membrane blebbing, the appearance of phosphatidylserine on the outer leaflet of the plasma membrane, and the activation of caspase-3.

**Conclusions/Significance:**

These results demonstrate that barnase is a potent toxic agent for targeting to cancer cells.

## Introduction

Barnase, a ribonuclease from *Bacillus amyloliquefaciens*, is synthesized as an active proenzyme, processed by the removal of the amino-terminal signal peptide, and secreted into the extracellular space. In this bacterial species, barstar, a specific intracellular inhibitor of barnase, is produced. Barstar tightly binds to barnase and thereby inhibits its intracellular enzymatic activity and protects host cells from the damaging effect of this RNase. Barnase is a small (110 aa) single-chain protein. It has no disulfide bonds and requires no post-translational modifications, divalent cations, or other non-peptide components for its function [1], [2]. Due to these favorable features, barnase is active in any cell type in which it is expressed. The ability of barnase to cleave RNA has been exploited in a wide variety of bio-applications since introduction of this enzyme into cells causes cell death. Specific ablation of particular cells is feasible by directing barnase gene expression via the use of cell-specific promoters [3]–[5]. Alternatively, proteins that target barnase to specific cells endow specificity to barnase action [6]–[8].

The toxic effect of barnase gene expression was used to design vectors for positive selection of cloned inserts [9], [10], to generate male and female sterility in plants [11], [12], to confer nematode resistance to crops [13], to produce potent agents for killing the third instar larvae of the cotton bollworm [14], to study the diseases caused by the loss of a specific cell type in mammals, and to eliminate cancer cells [5]. These examples clearly illustrate the effective and specific elimination of cells in different species by the use of barnase gene expression; however, little work has been focused on the effects of exogenous addition of barnase on malignant and normal mammalian cells. In fact, the examination of barnase nephrotoxicity is the only published example [15]. Therefore, the goal of this work was to characterize the effects of the ribonuclease barnase on human cancer and normal cells.

RNases are currently under intense investigation for their anticancer potential [16], [17]. The most promising among them are human pancreatic-type RNases well tolerated by the human immune system. But the cytotoxic potential of many of them is reduced by their sensitivity to inhibition by cytoplasmic ribonuclease inhibitor (RI) found in every mammalian cell studied [18]. Several approaches have been explored to reduce the sensitivity of pancreatic-type RNases to RI [19]–[22]. The use of RNases intrinsically resistant to RI provides another way to overcome this obstacle. We examined the susceptibility of barnase to RI and found that barnase is fortunately insensitive to inhibition by RI.

Cancer cells are a specific cell population, characterized by the presence of tumor-specific promoters and cancer markers. One of these markers is the HER2 antigen (also called HER-2, ERBB2, p185HER-2), which is overexpressed in a wide variety of human neoplasms [23], particularly in ovarian and breast carcinomas [24], [25]. We fused two barnase molecules to single-chain variable fragment (scFv) of the humanized antibody 4D5, which recognizes the extracellular domain of HER2, to produce scFv 4D5-dibarnase [8]. We made an immunoRNase (IR) that included two ribonucleases per carrier, since specific cytotoxicity is limited by the cell surface density of the HER2 antigen. This configuration enabled the introduction of twice the ribonuclease activity into cells with just one HER2 receptor. As shown previously [26], a two-fold increase in the number of RNase molecules in the immunoconjugate potentiated it by fifteen-fold. The purpose of this study was to examine whether scFv 4D5-dibarnase is able to interact specifically with HER2-positive human ovarian cells, be internalized into the cells, and exert cytotoxicity.

Consequently, in this work we estimated advantages of barnase for developing anticancer drugs and demonstrated the potency of barnase-based immunoRNase for cancer cell ablation.

## Results

### Characterization of barnase and scFv 4D5-dibarnase

**Figure 1:**
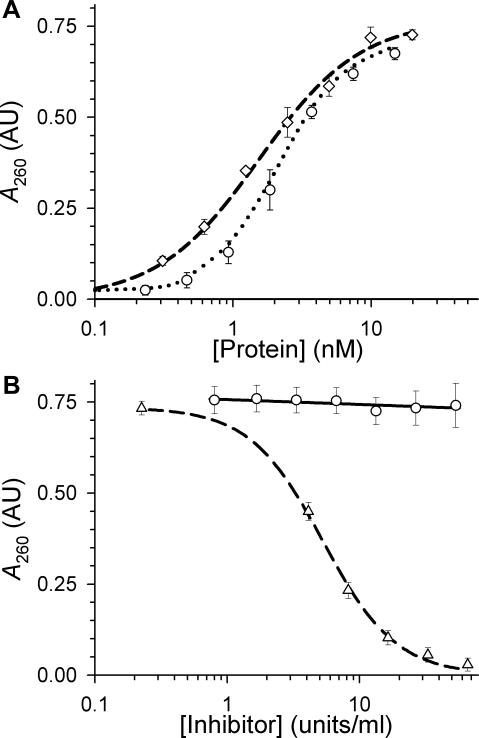
Ribonuclease activity assay. (A) The ribonuclease activities of barnase (dashed line and diamonds) and scFv 4D5-dibarnase (dotted line and circles) were determined according to the method of Rushizky et al. [58]. The x-axis represents the concentration of barnase alone or the half-concentration of scFv 4D5-dibarnase. The absorbance of 0.5 AU_260_ corresponds to the activity of 2 nM native barnase as previously described [27]. (B) Susceptibility of barnase to hRI (solid line and circles) and of scFv 4D5-dibarnase to barstar (dashed line and triangles). Data are means±SD of triplicate determinations; the curves are the results of sigmoid regression performed with SigmaPlot software.

Recombinant proteins were produced in *E. coli* and purified as described in Materials and Methods. The proteins obtained were of the expected size and homogenous according to SDS-PAGE (data not shown). The enzymatic activity of prepared barnase was 1.8×10^6^ units/mg, which was consistent with previously published values [27]. The ribonuclease activity of each barnase enzyme in the scFv 4D5-dibarnase fusion protein was 75% of native barnase (Figure 1A). The ribonuclease activity of scFv 4D5-dibarnase was inhibited by barstar (Figure 1B, dashed line). Thus, barnase moiety of scFv 4D5-dibarnase retained its functionality.

Following penetration into cells, the exogenously added RNase may fail to be active due to susceptibility to the cytoplasmic ribonuclease inhibitor [18]. Therefore, before testing the cytotoxicity of scFv 4D5-dibarnase, we examined the sensitivity of barnase to human ribonuclease inhibitor (hRI). At a concentration of four times greater than that required to inhibit RNase A by 50% (determined according to the manufacturer's instructions), hRI did not inhibit barnase (Figure 1B, solid line).

### Binding of barnase and scFv 4D5-dibarnase to cells

**Figure 2:**
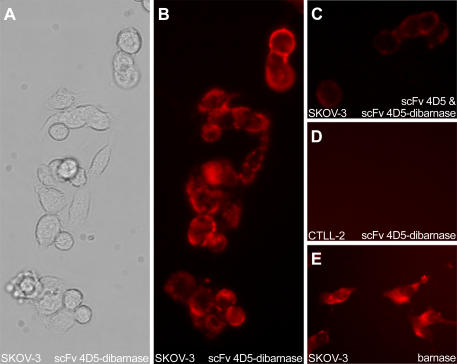
Binding of barnase and scFv 4D5-dibarnase to cells. The cell-binding ability of the recombinant proteins demonstrated by fluorescent microscopy. Cells were incubated at 4°C for 1 h with either 20 nM scFv 4D5-dibarnase (A, B and D), or a mixture of 20 nM scFv 4D5-dibarnase and 20 nM scFv 4D5 (C), or 20 µM barnase (E). Unbound proteins were removed, and then living (A–D) or fixed (E) cells were stained with rabbit anti-barnase antiserum and GAR-TR as described in Materials and Methods. The scFv 4D5-dibarnase bound to HER2-positive SKOV-3 cells (A and B), this specific binding was inhibited by scFv 4D5 (C). The scFv 4D5-dibarnase did not bind to HER2-negative CTLL-2 cells (D). Cytoplasmic staining of SKOV-3 cells with 20 µM barnase was observed (E). Magnification, 400×.

The binding of barnase and scFv 4D5-dibarnase to HER2-overexpressing human ovarian carcinoma SKOV-3 cells [28] and murine CTLL-2 cytotoxic T-cells lacking human HER2 was determined by fluorescent microscopy. The membrane fluorescence of SKOV-3 cells, but not CTLL-2 cells, stained with 20 nM scFv 4D5-dibarnase was observed (Figure 2, B and D). In controls, when scFv 4D5-dibarnase or rabbit anti-barnase antiserum were omitted, no fluorescence was detected in SKOV-3 cells and CTLL-2 cells (data not shown). The addition of scFv 4D5 to scFv 4D5-dibarnase led to notable quenching of cell membrane fluorescence (Figure 2, compare B and C), indicating that scFv 4D5-dibarnase bound to the HER2 receptor. No fluorescence was detected in either SKOV-3 cells or CTLL-2 cells in the presence of 20 nM barnase (data not shown). At barnase concentration of 20 µM, a bright cytoplasmic staining of SKOV-3 cells was observed (Figure 2E), suggesting penetration of barnase into cells. Appropriate controls without either barnase or rabbit anti-barnase antiserum were negative (data not shown).

**Figure 3:**
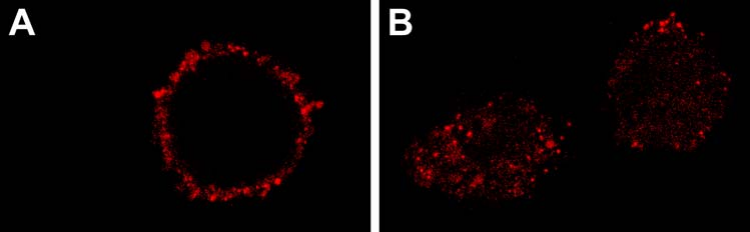
Binding and internalization of scFv 4D5-dibarnase in BT-474 cells visualized by confocal microscopy. (A) Cells were incubated with scFv 4D5-dibarnase at 4°C or (B) at 37°C. The scFv 4D5-dibarnase was detected with rabbit anti-barnase antiserum followed by GAR-PE. Fluorescence was observed predominantly on the surface of cells incubated at 4°C and inside the cells incubated at 37°C. This difference in the localization of the fluorescent label suggests internalization of scFv 4D5-dibarnase at 37°C in BT-474 cells.

The interaction of scFv 4D5-dibarnase with HER2-overexpressing human breast carcinoma BT-474 cells [25] was studied by confocal microscopy. BT-474 cells were incubated with 20 nM scFv 4D5-dibarnase at either 4°C to suppress internalisation or 37°C to let internalization and stained with rabbit anti-barnase antiserum followed by phycoerythrin-conjugated goat anti-rabbit IgG. The fluorescence was observed predominantly on the surface of the cells incubated at 4°C (Figure 3A) and inside the cells incubated at 37°C (Figure 3B), indicating that scFv 4D5-dibarnase binds to and penetrates into BT-474 cells. In controls, when scFv 4D5-dibarnase or rabbit anti-barnase antiserum were omitted, no fluorescence was detected in BT-474 cells (data not shown).

### Internalization of scFv 4D5-dibarnase investigated by electron microscopy

**Figure 4:**
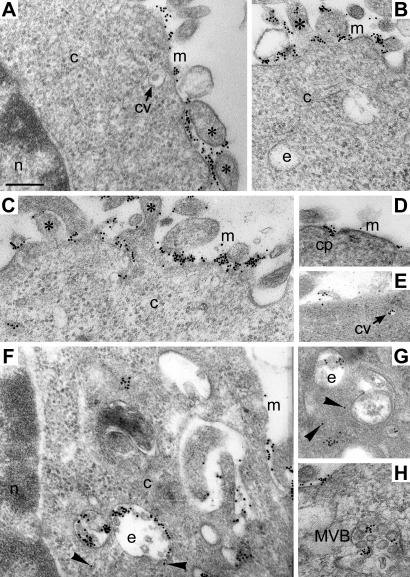
Internalization of scFv 4D5-dibarnase into HER2-positive SKOV-3 cells demonstrated by electron microscopy. SKOV-3 cells were incubated with 20 nM scFv 4D5-dibarnase-Au for 1 h at 4°C or at 37°C. (A and B) At 4°C, the gold label was deposited on the cytoplasmic membrane (m) and protrusions (asterisks) but not inside the cell. (C–H) At 37°C, scFv 4D5-dibarnase-Au bound to the cell surface in the same manner as at 4°C but was also found inside the cells in coated pits (cp) (D), coated vesicles (cv) (E), endosomes (e) (F and G), cytoplasm (c) (F and G, arrowheads), and multivesicular bodies (MVB) (H). The scFv 4D5-dibarnase-Au was not found in the nucleus (n) (F). Bar, 200 nm.

The intracellular localization of scFv 4D5-dibarnase was explored by electron microscopy. The scFv 4D5-dibarnase was complexed with gold particles (Au). The scFv 4D5-dibarnase-Au complex bound to SKOV-3 cells (Figure 4) but did not bind or penetrate CTLL-2 cells (data not shown) at 4°C and 37°C. Upon binding of the complex to the cell surface of SKOV-3 cells, the gold particles were deposited on protrusions and smooth parts of the cell membrane (Figure 4, A–D). The penetration of scFv 4D5-dibarnase-Au into SKOV-3 cells was observed at 37°C but not at 4°C, implying that penetration is a temperature-dependent process. The internalization of scFv 4D5-dibarnase-Au involved the formation of coated pits (Figure 4D) that budded from the cell membrane and transformed into coated vesicles (Figure 4E). Inside the cells, most of the gold particles were located in endosomes (Figure 4, F and G). A few gold particles were found free in the cytoplasm adjacent to the endosomes (Figure 4, F and G, arrowheads). These observations suggest that scFv 4D5-dibarnase may be released from endosomes into the cytoplasm. The gold particles also occurred in multivesicular bodies (Figure 4H). Nuclei were not labeled (Figure 4F).

### Effect of barnase and scFv 4D5-dibarnase on cell survival

**Figure 5:**
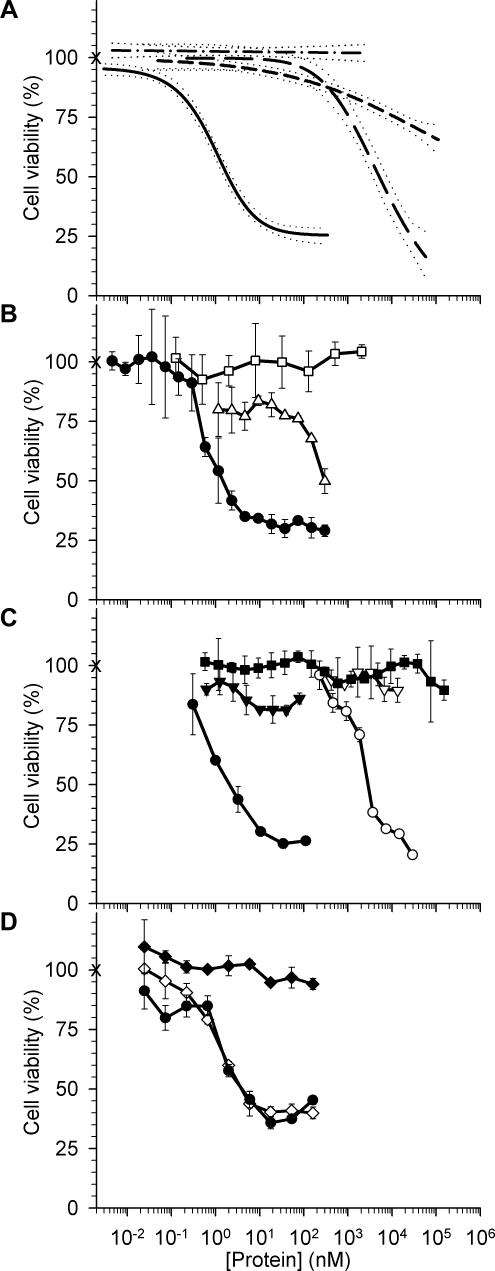
Effects of recombinant proteins on cell viability as determined by MTT assay. (A) The effects of barnase and scFv 4D5-dibarnase on the viability of human cancer and normal cells. SKOV-3 cells were treated for 72 h with barnase (long dashed line) or scFv 4D5-dibarnase (solid line), and hPBMCs were treated with barnase (short dashed line) or scFv 4D5-dibarnase (dashed-dotted line). (B) The competitive inhibition of scFv 4D5-dibarnase cytotoxicity by scFv 4D5. SKOV-3 cells were treated for 72 h with scFv 4D5-dibarnase in the absence (black circles) or presence (white triangles) of 300 nM scFv 4D5 or with scFv 4D5 alone (white squares). (C) The inhibition of barnase cytotoxicity and scFv 4D5-dibarnase cytotoxicity by barstar. SKOV-3 cells were treated for 72 h with barnase (white circles), barnase and equimolar amounts of barstar (white triangles), scFv 4D5-dibarnase (black circles), scFv 4D5-dibarnase with three-fold molar excess of barstar (black triangles), or barstar alone (black squares). (D) The effects of hRI on the cytotoxicity of scFv 4D5-dibarnase. SKOV-3 cells were treated for 72 h with either scFv 4D5-dibarnase in the absence of hRI (black circles), scFv 4D5-dibarnase in the presence of hRI (white diamonds), or hRI alone (black diamonds). Cell viability is expressed as the percentage of the metabolic activity of treated cells with respect to untreated cells (crosshair). Each regression curve in panel A (with 95% confidence intervals indicated by dotted lines) represents at least three independent experiments. Sigmoid regression was performed with SigmaPlot software. Curves in B–D represent typical experiments. Error bars (B–D) were obtained from triplicate measurements.

We investigated the effects of recombinant barnase and scFv 4D5-dibarnase on the survival of different human cancer cell lines. Human peripheral blood mononuclear cells (hPBMCs) were isolated from the peripheral blood of healthy donors and immediately used to examine the cytotoxicity of barnase and scFv 4D5-dibarnase on normal human cells. Murine CTLL-2 cytotoxic T-cells lacking human HER2 were also used. All cell lines and hPBMCs were incubated with proteins at various concentrations in complete culture media for 72 h and cell viability was evaluated in MTT assay (Table 1). The human breast carcinoma BT-474 cells demonstrated the highest sensitivity to barnase (IC_50_ = 0.21 µM), the lowest one was shown by the myelocytic leukemia HL-60 cells (IC_50_ = 82 µM). For other cancer cell lines, barnase was toxic with IC_50_ ranging from 2.4 to 13 µM. The dose-response curves did not reach saturation plateau, suggesting nonspecific interaction of barnase with cells. The SKOV-3 cells showed moderate sensitivity (IC_50_ = 5 µM), demonstrating more general response to the barnase-induced cytotoxicity than BT-474 cells. Therefore SKOV-3 cells were used for further characterization of the scFv 4D5-dibarnase effects on HER2-overexpressing cells. For hPBMCs, the maximal cytotoxic effect (27%) was achieved at 110 µM barnase (Figure 5A, short dashed line) while for SKOV-3 cells, 1.2 µM of barnase was sufficient to produce the same effect. Thus, barnase toxicity was two orders of magnitude greater for human cancer SKOV-3 cells than for hPBMCs.

**Table 1:** Cytotoxic activity of recombinant barnase and scFv 4D5-dibarnase proteins^1^.

Cells^2^	Type	barnase, nM			4D5 scFv-dibarnase-His_5_, nM		
		IC_30_	IC_50_	IC_70_	IC_30_	IC_50_	IC_70_
*SKOV-3^3^	ovarian carcinoma	1.5×10^3^	5.0×10^3^	18×10^3^	0.55	1.8	14
HEK293	embryonal kidney	3.0×10^3^	10×10^3^	46×10^3^	74	490	1.8×10^3^
*A-431	epidermoid carcinoma	2.5×10^3^	10×10^3^	63×10^3^	>740	-	-
*BT-474^3^	breast carcinoma	0.01×10^3^	0.21×10^3^	2.0×10^3^	0.72	2.4	9.2
MCF7	breast carcinoma	3.1×10^3^	13×10^3^	40×10^3^	>300	-	-
LOX	malignant melanoma	ND	ND	ND	>300	-	-
U-937	monocytic leukemia^5^	1.1×10^3^	2.4×10^3^	6.2×10^3^	>8.1×10^3^	-	-
HL-60	myelocytic leukemia^5^	36×10^3^	82×10^3^	>82×10^3^	>8.1×10^3^	-	-
K-562	erythrocytic-megakaryocytic leukemia^5^	5.8×10^3^	12×10^3^	31×10^3^	140	460	1×10^3^
CTLL-2	murine cytotoxic T-lymphocytes	>80×10^3^	-	-	64	>74	-
hPBMCs^4^	human peripheral blood mononuclear cells	>110×10^3^	-	-	>2.6×10^3^	-	-

ND, not determined; -, not reached

1 Cell viability assays were performed as described in Materials and Methods. The IC_nn_ is the concentration that results in nn% reduction of cell viability after 72 h of incubation with protein.

2 All the cells, except for CTLL-2, are of human origin; cell lines marked by asterisk were from American Type Cell Collection, the others were from cell collection of Department of Immunology, Shemyakin and Ovchinnikov Institute of Bioorganic Chemistry, Russia.

3 Cell lines overexpressing HER2.

4 hPBMCs were isolated from the peripheral blood of healthy donors.

5 Types of myeloid leukemia cell lines are termed according to [60].

Exposure of HER2-overexpressing SKOV-3 cells to scFv 4D5-dibarnase for 72 h demonstrated a dose-dependent cytotoxicity from 0.1 nM to 20 nM (Figure 5A, solid line). Further increases in the concentration enhanced the cytotoxicity slightly, pointing to that the effect of scFv 4D5-dibarnase was limited by the cell surface density of the HER2 receptor. The IC_50_ of scFv 4D5-dibarnase was 1.8 nM, which was 2800 times less than the IC_50_ of barnase (Figure 5A, compare solid and long dashed lines). The BT-474 cells showed the scFv 4D5-dibarnase sensitivity comparable to that of SKOV-3 cells. As IC_50_ and IC_30_ of scFv 4D5-dibarnase was 1.3 times higher for BT-474 cells than for SKOV-3 cells but IC_70_ was 1.5 times lower for BT-474 cells than for SKOV-3 cells (Table 1). Noteworthy, while effects of barnase alone on SKOV-3 and BT-474 cells differed 25 times, the scFv 4D5-dibarnase demonstrated similar effects on these HER2-positive cells. At the same time, HER2-negative hPBMCs were not affected by scFv 4D5-dibarnase at concentrations up to 2600 nM (Figure 5A, dashed-dotted line). These findings indicate that the effect of scFv 4D5-dibarnase was specific and receptor-mediated.

To further confirm that the cytotoxicity of scFv 4D5-dibarnase was mediated by the interaction of the scFv 4D5 moiety with the HER2 receptor, we examined the effect of scFv 4D5-dibarnase on SKOV-3 cells in the presence of scFv 4D5 at a concentration of 300 nM. While scFv 4D5 itself did not affect SKOV-3 cells (Figure 5B, white squares) at concentrations of 0.1 nM to 2000 nM, the miniantibody diminished the cytotoxicity of scFv 4D5-dibarnase in a dose-dependent manner (Figure 5B, white triangles). This dependence suggests that scFv 4D5 and scFv 4D5-dibarnase compete for the same binding site and further confirmed the specific interaction of scFv 4D5-dibarnase with the HER2 receptor.

To determine whether the enzymatic activity of barnase was essential for cytotoxicity, barstar, a specific inhibitor of barnase, was utilized. Barstar alone did not inhibit the viability of SKOV-3 cells at concentrations up to 2000 nM. (Figure 5C, black squares). Addition of barstar to barnase at equimolar amounts abolished barnase cytotoxicity at a concentration range 0.4–13 µM (Figure 5C, white triangles). When added in three-fold excess, barstar reduced the toxic effect of scFv 4D5-dibarnase at concentrations of 0.6 nM to 80 nM (Figure 5C, black triangles).

The inhibition of scFv 4D5-dibarnase cytotoxicity by barstar and scFv 4D5 confirmed that both 4D5 scFv and barnase contribute to the cytotoxicity of scFv 4D5-dibarnase to SKOV-3 cells.

To test whether hRI influences the effects of scFv 4D5-dibarnase on cancer cells, hRI and scFv 4D5-dibarnase were incubated at a ratio of 100 units hRI to 1 µg scFv 4D5-dibarnase for 30 min at 4°C and then were added to the cells. The hRI alone neither influenced SKOV-3 cell survival (Figure 5D, black diamonds) nor scFv 4D5-dibarnase cytotoxicity (Figure 5D, compare black circle and white diamonds).

### RNA degradation induced by barnase in SKOV-3 cells

**Figure 6:**
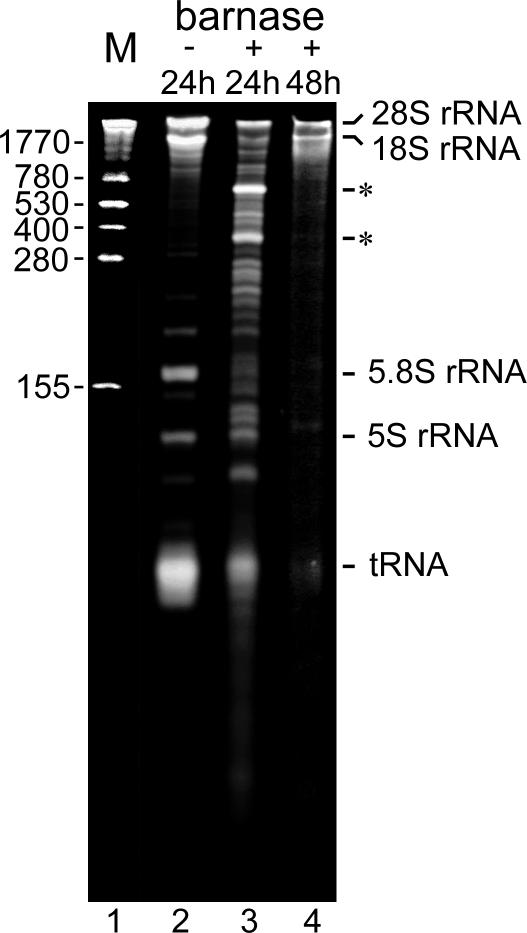
Cellular RNA undergoes degradation in SKOV-3 cells treated with barnase. SKOV-3 cells were exposed to 50 µM barnase for 24 h (lane 3) or 48 h (lane 4). Total RNA was isolated as described in Materials and Methods and analyzed on a 9% polyacrylamide gel containing 7.5 M urea. Each sample lane was loaded with RNA from 2×10^5^ treated (+) or untreated (−) cells. Lane 2 corresponds to mock-treated control. The positions of the RNA molecular weight standards (lane 1) are shown as the number of bases to the left of panel. Asterisks indicate the most prominent bands that appear as a result of enzymatic cleavage of high molecular weight rRNA by barnase (lane 3).

Polyacrylamide gel analysis of total cellular RNA isolated from SKOV-3 cells demonstrated that cellular RNA undergoes degradation in cells treated with 50 µM barnase (Figure 6). Extensive RNA degradation was evident 24 h after exposure of cells to barnase (lane 3); after 48 h, degradation of cellular RNA was nearly complete (lane 4). Both low molecular weight tRNA and 5.8S rRNA, but not 5S rRNA, seemed more susceptible to 24-h barnase treatment. The appearing of the additional bands (lane 3, asterisks) indicates the enzymatic cleavage of high molecular weight rRNA by barnase. Digital image analysis of the gel in Figure 6 (lanes 2 and 3) shows that the relative abundance of tRNA and 5.8S rRNA was decreased to 30% and 16% of control cells, respectively, while levels of 5S, 18S, and 28S rRNA were decreased to 60%, 43%, and 54% of control cells, respectively.

### Mechanism of action of barnase and scFv 4D5-dibarnase on SKOV-3 cells

**Figure 7:**
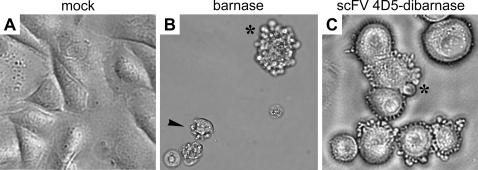
SKOV-3 cells treated with either barnase or scFv 4D5-dibarnase for 72 h demonstrated membrane blebbing. (A) The mock-treated cells were attached to the plate and were flat. (B and C) Cells incubated with either 50 µM barnase or 50 nM scFv 4D5-dibarnase became rounded and detached. Membrane blebbing (B and C, asterisks) and disrupted cells (B, arrowhead) were observed. Phase-contrast microscopy of a random field at a magnification of 400×.

To identify and characterize the mode of cell death triggered by barnase or scFv 4D5-dibarnase treatment, SKOV-3 cells were prepared as described in Materials and Methods. Both barnase and scFv 4D5-dibarnase induced a characteristic apoptotic blebbing of the cellular membrane that was detectable in certain cells as early as 6 h after treatment and continued to be evident for the following 72 h (Figure 7).

**Figure 8:**
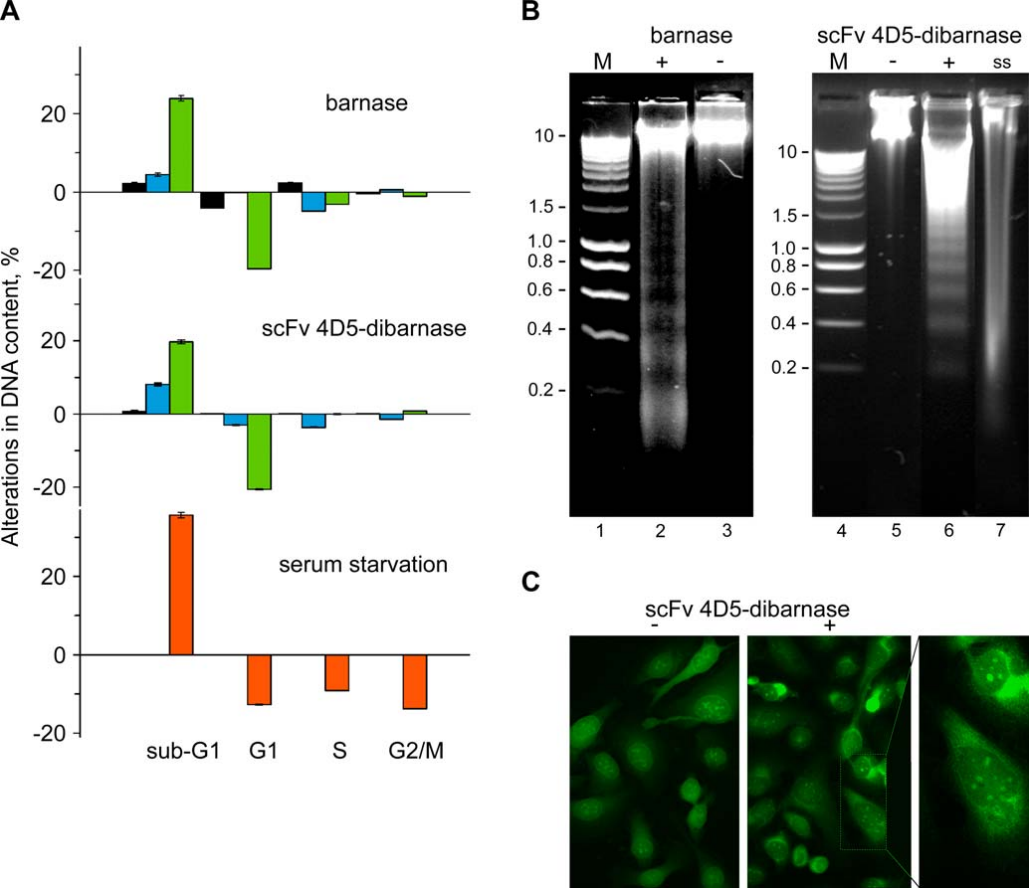
Barnase and scFv 4D5-dibarnase cause DNA fragmentation in SKOV-3 cells. (A) Flow cytometric analysis of the cell cycle distribution was performed as described in Materials and Methods. Histograms represent the differences in the percentages of cells between barnase- or scFv 4D5-dibarnase-treated and untreated cells for each cell cycle stage (sub-G1, G1, S, and G2/M) measured after 24 h (black bars), 48 h (blue bars), and 72 h (green bars) of treatment. Error bars show the standard deviation. Positive controls for DNA fragmentation were SKOV-3 cells cultured for 7 days in serum-free medium (orange bars). (B) DNA electrophoresis assay. Cells were treated with either 50 µM barnase or 50 nM scFv 4D5-dibarnase. Seventy-two hours later, genomic DNA of both treated (+) and untreated (−) cells was isolated and DNA from equal numbers of cells was resolved in non-denaturing 1.5% agarose gels. The DNA was visualized by ethidium bromide staining. Chromatin fragments resulting from internucleosomal cleavage were present in samples of DNA from cells treated with barnase (lane 2) and scFv 4D5-dibarnase (lane 6). DNA of serum-starved (ss) cells were cleaved irregularly (lane 7). Lanes 3 and 5 represent untreated controls. Lanes 1 and 4 are molecular weight markers ((M) HyperLadder I, Bioline). (C) Cells were exposed to 50 nM scFv 4D5-dibarnase for 72 h and then stained with acridine orange, analyzed by fluorescence microscopy, and photographed. A representative case of nuclear pyknosis and fragmentation (karyorrhexis) is shown (inset). Magnification, 400× (1200×, inset).

DNA fragmentation in dying cells is a general end point that is common to both necrotic and apoptotic mechanisms of cell death. To determine whether barnase and scFv 4D5-dibarnase induce DNA breaks in SKOV-3 cells, propidium iodide (PI) stained cells were analyzed for alterations in cell cycle distribution using DNA content measurements via flow cytometry. Treatment of SKOV-3 cells with either barnase or scFv 4D5-dibarnase resulted in a gradual elevation of the proportion of cells in the sub-G1 phase (cells containing less DNA than 2N), compared with untreated control cells (Figure 8A). Barnase-treated SKOV-3 cells showed increases of 2.1%, 4.5%, and 23.9% in the number of cells in sub-G1 phase and decreases in 4.0%, 0.2%, and 19.5% in the number of cells in G1 phase of the cell cycle compared with controls after 24, 48, and 72 h of treatment, respectively. Similarly, scFv 4D5-dibarnase-treated SKOV-3 cells showed increases of 8.5%, 8.1%, and 19.7% in the number of cells in sub-G1 phase and decreases of 7.5%, 3.0%, and 20.6% in the number of cells in G1 phase compared with their respective controls. In addition, the number of cells in S phase was decreased by 4.9% and 3.1% in barnase-treated cells after 48 and 72 h, respectively, and by 3.6% in scFv 4D5-dibarnase-treated cells after 48 h. On the other hand, cell cycle analysis revealed no significant differences in cells in G2/M stage of the cell cycle between control and treated cells (Figure 8A). These results suggest that both barnase and scFv 4D5-dibarnase induced DNA breaks predominantly in G1 cells. In contrast, serum-starved SKOV-3 cells exhibited an increase in the percentage of cells in sub-G1 phase (35.7%), accompanying decreases in the other three phases [G1 (12.7%), S (9.1%), and G2/M (13.8%)] compared with control cells.

To elucidate which apoptotic or necrotic mode of DNA fragmentation was triggered by barnase and scFv 4D5-dibarnase, genomic DNA that was isolated from SKOV-3 cells treated with either 50 µM barnase or 50 nM scFv 4D5-dibarnase was electrophoresed through a 1.5% agarose gel (Figure 8B). Seventy-two hours after barnase or scFv 4D5-dibarnase treatment, SKOV-3 cells displayed characteristic internucleosomal chromatin cleavage (Figure 8B, lanes 2 and 6), differing from the irregular DNA cleavage of serum-starved SKOV-3 cells (Figure 8B, lane 7). Furthermore, treatment of SKOV-3 cells with scFv 4D5-dibarnase induced a distinct pattern of nuclear pyknosis and fragmentation (karyorrhexis) as observed by fluorescence microscopy after staining of cells with acridine orange (Figure 8C).

**Figure 9:**
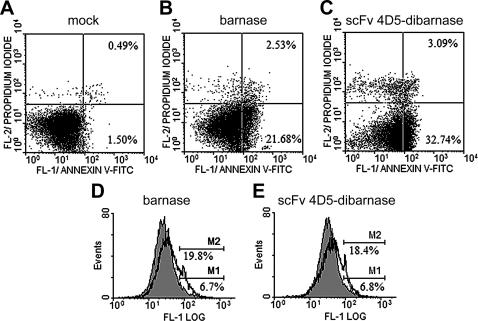
Barnase and scFv 4D5-dibarnase induced apoptosis accompanied by phosphatidylserine externalization and caspase-3 activation. (A–C) SKOV-3 cells were mock-treated (A) or treated with either 50 µM barnase (B) or 50 nM scFv 4D5-dibarnase (C) for 72 h. Cells were analyzed for early apoptosis by Annexin-V-FITC/PI staining. The lower left quadrants of each panel show the viable cells, which exclude PI and are negative for Annexin-V-FITC binding. The upper right quadrants contain the non-viable, necrotic cells, which are positive for both Annexin-V-FITC binding and PI uptake. The lower right quadrants represent apoptotic cells, Annexin-V-FITC positive and PI negative. One representative experiment out of three is shown. (D and E) Caspase-3-like enzymatic activities of cells treated with either 50 µM barnase (D, unfilled peak) or 50 nM scFv 4D5-dibarnase (E, unfilled peak) for 72 h were assessed by the cleavage of the fluorogenic substrate PhiPhiLux-G_1_D_2_ and compared with that of untreated cells (filled peaks). M1 and M2 markers correspond to levels of caspase-3 activation in untreated and treated cells, respectively.

We also used Annexin-V-FITC/PI staining to measure the appearance of phosphatidylserine, a marker of apoptosis, on the outer leaflet of the plasma membrane of SKOV-3 cells (Figure 9, A–C). Cells treated for 72 h with either 50 µM barnase or 50 nM scFv 4D5-dibarnase were found to be Annexin-V-FITC positive and PI negative at a higher percentage (21.7% and 32.7%, respectively) than in untreated cells (1.5%). These results indicate that the nature of the cell death induced by both barnase and scFv 4D5-dibarnase is apoptotic. An increase in necrotic cells (Annexin-V-FITC positive and PI positive) was 2.0% for barnase-treated cells and 2.6% for scFv 4D5-dibarnase-treated cells compared with controls, ten-fold less than that for apoptotic cells.

To further investigate the mode of cell death induced by barnase and scFv 4D5-dibarnase, we measured the activation of an apoptosis-specific caspase-3 by proteolytic cleavage assay of PhiPhiLux-G_1_D_2_ substrate (Figure 9, D and E). Caspase-3-like activity was increased by 13.1% for barnase- and by 11.6% for scFv 4D5-dibarnase-treated SKOV-3 cells compared with untreated controls.

In conclusion, the ability of barnase and scFv 4D5-dibarnase to induce membrane blebbing, the appearance of phosphatidylserine on the outer leaflet of the plasma membrane, internucleosomal chromatin fragmentation, and the activation of caspase-3 support the notion that these proteins trigger apoptotic cell death.

## Discussion

Barnase has been successfully employed in a number of studies for the removal of cells in various species [3]–[5 and 9–14]; however, the cytotoxic effects of barnase on cancer cells have not been investigated sufficiently. Here, recombinant barnase was shown to be toxic to human carcinoma cell lines with IC_50_ values ranging from 0.2 to 13 µM and to leukemia cell lines with IC_50_ values ranging from 2.4 to 82 µM (Table 1). Compared with other RNases [29], barnase is moderately toxic to human cancer cells. The effects of barnase on different cancer cell lines varied 400-fold. The most sensitive cell line was BT-474 (IC_50_ = 0.21 µM), and the least sensitive one was HL-60 (IC_50_ = 82 µM). The wide spread in the IC_50_ values for various cells lines is also inherent to the bovine seminal ribonuclease (BS RNase) and onconase, a ribonuclease from *Rana pipiens*. The effects of BS RNase on carcinoma cell lines varied 570-fold; and effects of onconase on carcinoma and leukemia cell lines varied 6000-fold [30]. The observed variety in the susceptibility of cell lines to barnase can be caused by differences in modifications and/or composition of the cell surface molecules which determine the binding of barnase to the cell surface. The manner and strength of the binding influence the efficiency of cellular uptake of RNase [31] or the internalization pathway of RNase. This pathway, which determines the intracellular location and, ultimately, the access to the RNA substrate, was shown to vary in malignant and normal cells for the same RNase [32].

To influence cell survival, barnase must first interact effectively with the cell membrane. The cell-binding ability of RNases correlates with their net positive charge [33]. The high positive net charge of barnase (pI ∼9) [34] aids its binding to the negatively charged cell membrane. Cytoplasmic fluorescence was observed in cells stained with barnase, indicating that barnase penetrated the cells. Following penetration into the cytoplasm, the RNase would be able to effectively cleave RNA provided that the RNase is resistant to RI, a ribonuclease inhibitor that has been shown to exist in all mammalian cells [18]. We found that barnase was not inhibited by hRI. This finding is consistent with data for other members of the N1/T1 ribonuclease family, such as RNase Sa and RNase Sa3 [35], RNase T1, and ribonuclease U1 [36], which are unaffected by RI. This ability to escape hRI increases the cytotoxic potential of barnase. Besides, barnase possesses the conformational stability (melting temperature, Tm = 54°C) that resembles that of the highly toxic G88R mutant of RNase A (Tm = 60°C) [37]. The stability of the RNase is essential for cytotoxicity since its structure determines the resistance of the protein to intracellular proteases. The catalytic activity of barnase [10.9×10^6^ M^−1^s^−1^, poly(I)] [38] markedly exceeds the activity of many RNases and, in particular, that of the highly toxic onconase [1.4×10^3^ M^−1^s^−1^, poly(C)] [37]. Thus, a high positive net charge, resistance to RI, conformational stability, and high catalytic activity are intrinsic properties of barnase that are especially suited for cytotoxicity.

An examination of RNA integrity in cells treated with barnase demonstrated serious RNA degradation, suggesting that barnase preserved its enzymatic activity in cells, interacted with cellular RNA, and hydrolyzed it. The abrogation of barnase cytotoxicity by barstar observed here supports the hypothesis that the cytotoxicity of barnase is caused by its ribonuclease activity. Furthermore, a mutant barnase lacking catalytic activity failed to induce toxicity in isolated perfused kidney [15]. From our results, barnase did not display a clear preference towards certain cellular RNA species. This finding is consistent with the preference of barnase for purine bases; however, barnase does not recognize specific nucleotide sequences and its base preference is less pronounced in polynucleotide substrates [38]. Among the well-characterized *in vivo* members of the RNase A superfamily, BS RNase predominantly cleaves 18S and 28S rRNA [39] and onconase cleaves primarily tRNA [40]. In fact, the inhibition of protein synthesis by onconase correlates with the dose which results in rRNA degradation [41].

Interestingly, barnase in the MTT assay proved to be selectively cytotoxic for cancer cells. Indeed, 100 µM barnase inhibited the survival of cancer cells (except HL-60 cells) by more than 70% while it inhibited the survival of hPBMCs by only 26%. Selectivity toward cancer cells was also reported for BS RNase [42] and for binase [43], a ribonuclease from *Bacillus intermedius* that shares 82% amino acid identity with barnase [44]. As barnase, these RNases have high positive net charges [29]. Cancer cells expose more negatively charged phospholipids [45] as well as more glycolipids and glycoproteins [46], [47] on the outer plasma membrane than do normal cells. This feature may be one of the reasons for the observed selectivity of cationic RNases and, in particular, of barnase toward cancer cells. Another noteworthy fact is that barnase exhibited the lowest toxicity to HL-60 cells, which proliferated more slowly than other human cancer cells tested. Similarly, onconase was shown to preferentially kill actively proliferating cells [48]. The rapid proliferation of cancer cells could make them more reliant on the integrity of their RNA.

To endow specificity to barnase action we previously fused it to single-chain variable fragment of 4D5 antibody recognizing extracellular domain of HER2 receptor [8]. Here, we evaluated the effects of anti-HER2 immunoRNase scFv 4D5-dibarnase on human cells.

ERB-hRNase, an immunoRNase based on RI-sensitive human pancreatic RNase, has been satisfactory tested on HER2-positive carcinoma cells. The cytotoxicity displayed by ERB-hRNase could be presumably explained by the steric hindrance of the interaction between hRNase and RI caused by the ERB antibody moiety. Recently, the authors have found out that ERB-hRNase is inhibited by RI, but the level that this IR reaches in the cytosol neutralizes RI [49]. Having chosen RI-resistant barnase as a toxin moiety, we produced the first IR targeted to HER2 and benefiting in cytotoxicity from its RI-resistance.

Analysis of the functional activity of scFv 4D5-dibarnase revealed that the antibody moiety kept the ability to bind to HER2 [8]. We found that each barnase in the fusion protein retained 75% of activity of barnase alone, similar to results reported for another IR targeted to HER2 [50]. The ribonuclease activity of scFv 4D5-dibarnase was inhibited by barstar, suggesting that barnase in this IR retained its ability to bind barstar.

We have shown that scFv 4D5-dibarnase bound to HER2-positive cells and that this binding was inhibited by scFv 4D5. The scFv 4D5-dibarnase penetrated HER2-positive SKOV-3 cells through coated pits and coated vesicles. This internalization was temperature-dependent. The scFv 4D5-dibarnase did not bind or penetrate HER2-negative cells. Taken together, these results suggest that scFv 4D5-dibarnase interacts with HER2 receptor and penetrates cells via specific receptor-mediated endocytosis. The majority of internalized scFv 4D5-dibarnase was found in endosomes, presumably from which the IR was released into the cytoplasm where it reached the RNA substrate and caused cytotoxicity.

The cytotoxicity of anti-HER2 targeted barnase to SKOV-3 cells was three orders of magnitude greater than barnase alone. A comparable increase was observed for RNase A targeted with transferrin or different antibodies to cells expressing transferrin receptor [26], [51]. The specific barnase inhibitor barstar abrogated this cytotoxic action while hRI did not. Consequently, the ribonuclease activity of scFv 4D5-dibarnase is essential for its cytotoxicity.

While the effects of barnase alone on SKOV-3 and BT-474 cells differed 25 times, scFv 4D5-dibarnase demonstrated similar specific cytotoxicity to both HER2-overexpressing cell lines, what agrees with comparable expression levels of HER2 receptor demonstrated immunohistochemically for these cell lines [52]. The cytotoxicity of scFv 4D5-dibarnase corresponded to HER2 expression levels of the cell lines tested. In particular, SKOV-3 cells, which transcribe approximately 60 times more HER2 mRNA than do HEK293 cells [53], [54], were 270 times more susceptible to scFv 4D5-dibarnase. Similar correlations were also observed for SKOV-3 and K-562 cell lines (Table 1). Having no effect on cell viability the scFv 4D5 diminished the cytotoxicity of scFv 4D5-dibarnase to HER2-positive cells in a dose-dependent manner. These results confirm that the action of scFv 4D5-dibarnase is receptor-mediated. Furthermore, the scFv 4D5-dibarnase did not exhibit cytotoxic effects on hPBMCs even at 2600 nM whereas this IR was able to reduce SKOV-3 cell survival by 50% at 1.8 nM. Thus, scFv 4D5-dibarnase demonstrated a greater than 1400-fold specificity toward HER2-overexpressing cancer cells compared with normal cells.

The IC_50_ values of different RNases targeted to various human carcinomas ranged from 0.3 nM to 1000 nM [30]. For instance, in HER2-overexpressing breast carcinoma SK-BR-3 cells, the IC_50_s of hERB-hRNase and ERB-HPR, fusion proteins consisting of human pancreatic RNase and scFv antibodies for specific targeting to HER2, were 12.5 nM and 50 nM, respectively [55], [50]. Taken together, these data suggest that scFv 4D5-dibarnase is a highly cytotoxic immunoRNase.

To identify and characterize the mode of cell death triggered by barnase and scFv 4D5-dibarnase, we determined whether protein-treated cells display characteristic features of apoptosis. SKOV-3 cells treated with either barnase or scFv 4D5-dibarnase displayed membrane blebbing, the appearance of phosphatidylserine on the outer leaflet of the plasma membrane, internucleosomal chromatin fragmentation, and the activation of caspase-3, suggesting that both proteins induce apoptosis.

We examined alterations in the cell cycle distribution of SKOV-3 cells in asynchronous culture and found that cells in G1 phase were more susceptible to treatment with barnase and scFv 4D5-dibarnase compared with cells in other phases. Cells in G1 phase actively synthesize RNA for their further transition to S phase. For this reason, the hydrolysis of cellular RNA by barnase inhibited the G1 to S phase transition (antiproliferative effect), which was accompanied by a decrease in the amount of cells in S phase. However, this effect did not lead to the accumulation of G1 phase cells since these cells underwent apoptosis (cytotoxic effect of barnase). While one should not exclude the induction of apoptosis in S phase cells since the amount of G2/M phase cells were unaltered compared with untreated cells, barnase did not likely inhibit the S- to G2/M- and to G0/G1- phase transitions. Since the effect of barnase depends on the particular cell cycle stage, apoptosis is prolonged in asynchronous culture. The presence of the hallmarks of both early and late apoptosis in treated cells supports the latter statement.

Cell death through apoptosis was shown for a number of other proteins with RNase activity, such as onconase [56], binase [43], and the immunoRNase hERB-hRNase [49]. The most interesting property of RNases is their potential use as non-mutagenic alternatives to the harmful DNA-damaging cancer chemo- and radiotherapies.

Based on the effectiveness and selectivity of the cytotoxic effects of scFv 4D5-dibarnase on target cancer cells, we present a potentially valuable tool for cancer immunotherapy. Thus, here we demonstrated that the scFv 4D5-dibarnase fusion protein is successful example of the specific targeting of barnase to cancer cells. The efficacy of scFv 4D5-dibarnase should be further evaluated against human tumor xenografts in mice. The fusing of barnase with scFvs recognizing various cancer markers will enable the generation of new promising anticancer immunoRNases.

## Materials and Methods

### Materials

The following materials were used: torula yeast RNA (Boehringer Mannheim, Germany); human ribonuclease inhibitor (hRI; Promega); chromatographic media and columns (Amersham Biosciences); Ni-NTA Agarose (QIAGEN AG, Switzerland); lysozyme (Biolar, Russia); RPMI-1640 medium (PanEco, Russia); X-VIVO 15 medium (Cambrex); fetal calf serum (FCS; HyClone, Belgium); L-glutamine (Flow Laboratories, UK); recombinant interleukin-2 (IL-2; Biotech, Russia); phosphate buffered saline (PBS; PanEco, Russia); goat serum (DakoCytomation); phycoerythrin-conjugated goat anti-rabbit IgG (GAR-PE) (Santa Cruz Biotechnology); RNA molecular weight standard 0.16–1.77 kb (GibcoBRL); DNA molecular weight marker HyperLadder I (Bioline); RNase A (Fermentas, Lithuania); fluorescein isothiocyanate (FITC)-labbelled Annexin V and propidium iodide (Molecular Probes); PhiPhiLux-G_1_D_2_ (OncoImmunin, Inc., Gaithersburg, MD). Isopropylthio-β-D-galactoside (IPTG), 3-(4,5-dimethylthiazol-2-yl)-2,5-diphenyltetrazolium bromide (MTT), Texas Red-conjugated goat anti-rabbit polyclonal antibodies (GAR-TR), acridine orange, bovine serum albumin (BSA), saponin, and other chemicals were obtained from Sigma. Rabbit anti-barnase antiserum was kindly provided by Dr. R. W. Hartley from the National Institutes of Health, Bethesda, Maryland, USA.

### Protein expression and purification

Wild-type barnase was produced in *E. coli* strain TG1 transformed with the pPBa (provided by Dr. A. A. Schulga) plasmid that was developed for high-yield thermoinducible expression of barnase. Barnase was extracted and purified from culture media following the method of Hartley [27] with slight modifications. The protocol was scaled down to a 10-l culture in shaking flasks. Also, acetic acid was used instead of sulfuric acid during the acidification step. The mutant barstar C40/82A (here referred to as barstar) was expressed in *E. coli* strain HB101 carrying the plasmid pMT643 (a gift of Dr. R. W. Hartley). The protein was isolated from the supernatant of sonicated bacteria. The supernatant was fractionated with ammonium sulfate. The fraction of 35–70% saturation of ammonium sulfate was purified by gel-filtration on a Sephacryl S-200 HP (XK-16/100) column. Next, an anion exchange chromatography was performed on a Q-Sepharose FF column.

The plasmid encoding scFv 4D5 with a His_5_ tag (provided by Prof. A. Plückthun) was used to produce scFv 4D5 as described in [57]. The scFv 4D5-dibarnase with a C-terminal His_5_ tag was produced in *E. coli* strain SB536 (provided by Prof. A. Plückthun) transformed with plasmid pSD-4D5 scFv-dibarnase-His_5_ [8]. The bacteria were grown on selective agar with 1% glucose and were transferred to YTPS media (1% triptone, 1% yeast extract, 45 mM K_2_HPO_4_, 5 mM KH_2_PO_4_, 0.1 M NaCl, and 100 mg/ml ampicillin) supplemented with 1% glucose and cultivated for 12 h at 28°C. This culture, which typically reached 6 absorbance units (AU) at 550 nm, was diluted to an A_550_ = 0.17 AU in YTPS media with 2 mM MgCl_2_ and cultivated further to an A_550_ = 1.2 AU. Then, scFv 4D5-dibarnase expression was induced with the addition of 1 mM IPTG. The bacteria were then grown at 26°C for 12 h. Periplasmic proteins from harvested bacteria were extracted with 0.5 mg/ml lysozyme in TBS (30 mM Tris, 20 mM KH_2_PO_4_, 0.15 M NaCl, pH 7.8) by gentle mixing on ice for 40 min. The extract was clarified by centrifugation and supplemented with 0.05% Tween 20, 10% glycerol, 0.25 M NaCl, and 20 mM imidazole. The pH was adjusted to 7.8 at 4°C. The extract was applied to a column containing Ni-NTA Agarose and the column was washed first with high salt buffer (25 mM Tris, 20 mM KH_2_PO_4_, 0.5 M NaCl, 20 mM imidazole, 0.1% Tween 20, 10% glycerin, pH 7.8) and then with LSW buffer (25 mM Tris, 20 mM KH_2_PO_4_, 0.1 M NaCl, 10% glycerin, pH 7.8). Barstar that had bound scFv 4D5-dibarnase was removed via washing with 6 M guanidinium hydrochloride in LSW, and the protein bound to the column was refolded with a ten column volume (CV) linear gradient of guanidinium hydrochloride (6–0 M) in LSW at 3 cm/h. Then, the scFv 4D5-dibarnase was eluted with a 10 CV linear gradient of imidazole (0–0.2 M) in LSW at 3 cm/h. The peak corresponding to 0.1 M imidazole was pooled, ultrafiltered, and equilibrated on a PD10 column in storage buffer (25 mM K_2_HPO_4_, 23 mM MOPS, 0.1 M NaCl, 10 mM EDTA, 40% glycerin, pH 7.4).

The purity of all prepared recombinant proteins was verified by SDS-PAGE. Protein concentrations were determined by absorbance at 280 nm using the following extinction coefficients: 101407 for scFv 4D5-dibarnase (54 kDa), 49410 for scFv 4D5 (28 kDa), 26030 for barnase (12.4 kDa), and 20910 for barstar C40/82A (10.2 kDa).

### Ribonuclease activity assay

A ribonuclease activity of barnase and scFv 4D5-dibarnase was tested by the acid-insoluble RNA precipitation assay described in [58] on yeast RNA, but all volumes were scaled down five-fold. To determine whether hRI was able to inhibit barnase, serial two-fold dilutions of hRI (starting at 80 units) were preincubated with 90.5 ng (7.3 pmol) barnase and were then used in the Rushizky assay. The manufacturer (Promega) defined one unit of hRI as the amount that inhibits the activity of 5 ng (0.365 pmol) RNase A by 50%. To determine whether scFv 4D5-dibarnase was inhibited by barstar, scFv 4D5-dibarnase at a constant concentration of 12.5 nM was incubated with serial two-fold dilutions of barstar (maximum dose 315 nM). These mixtures were then used in the Rushizky assay. One unit of barstar is defined as an equimolar barnase amount [59].

### Cells and incubation conditions

Human peripheral blood mononuclear cells (hPBMCs) were isolated from blood of healthy donors by centrifugation through Ficoll-Paque PLUS (GE Healthcare, UK) and immediately used to examine the cytotoxicity of proteins. The cell lines listed in Table 1 (except hPBMCs) were maintained in RPMI-1640 medium supplemented with 10% FCS and 2 mM L-glutamine. Medium for CTLL-2 contained 100 units/ml IL-2. Cells were incubated in a 5% CO_2_ atmosphere at 37°C. The myeloid leukemia cell lines listed in Table 1 were termed according to [60].

### Determination of the binding by fluorescence microscopy

Cells were incubated in PBA (PBS with 1% BSA) with either barnase (20 nM or 20 µM), or 20 nM scFv 4D5-dibarnase, or a mixture of scFv 4D5-dibarnase and scFv 4D5 at a final concentration of 20 nM each for 1 h at 4°C. Next, these cells were incubated with secondary rabbit anti-barnase antiserum (1∶500) and then with GAR-TR (1∶1000) for 1 h at 4°C. After each incubation, the cells were washed twice with ice-cold PBS. For controls, either recombinant proteins, or rabbit anti-barnase antiserum, or both were omitted. To study barnase interaction with cells, SKOV-3 cells were incubated in PBA with 20 µM barnase for 1 h at 4°C. These cells were then washed with PBS three times and fixed with 2% paraformaldehyde in PBS for 30 min at room temperature (RT). The cell membranes were permeabilized in PBS containing 0.2% saponin and 5% goat serum for 20 min at RT. Cells were then stained at RT in the same solution with the antibodies as specified above. Cells were analyzed using an inverted fluorescence microscope Axiovert 200 (Zeiss, Germany). Images were captured using a CCD camera (AxioCam HRc, Zeiss, Germany) and AxioVision software (Zeiss, Germany). Images were further processed using Adobe Photoshop software (Adobe Systems, Mountain View, CA).

### Determination of the binding and internalization of scFv 4D5-dibarnase in cells by confocal microscopy

Cells were grown to 60% confluency in the Lab-Tek II chamber slide system (Nalge Nunc International), incubated with 20 nM scFv 4D5-dibarnase at 4°C for 1 h, washed, and incubated for 30 min at either 4°C or 37°C. Cells then were washed, fixed, and permeabilized as described in [61]. The scFv 4D5-dibarnase was detected with rabbit anti-barnase antiserum (1∶500) followed by GAR-PE (1∶400). After incubation with the antibodies at 4°C, 1 h each, the cells were washed twice with ice-cold PBS. For controls, either recombinant proteins, rabbit anti-barnase antiserum, or both were omitted. The slides were analyzed with a Nikon Eclipse TE2000 confocal microscope.

### Electron microscopy

Colloidal gold particles (10 nm) were prepared as described [62], complexed with scFv 4D5-dibarnase molecules, and purified in accordance with the recommendation of Slot and Geuze [63]. Cells were incubated in PBA with 20 nM scFv 4D5-dibarnase-gold complex (scFv 4D5-dibarnase-Au) for 1 h at either 4°C or 37°C. Then, the cells were washed twice with PBS, collected by centrifugation, fixed with 2% glutaraldehyde in PBS for 2 h, and post-fixed in 1% osmium tetroxide for 1 h. After dehydration in a series of increasing concentrations of ethanol, cells were embedded in Epon-Araldite according to a standard procedure. Ultrathin sections were stained using aqueous uranyl acetate and lead citrate and then were examined using a JEOL 100 CX electron microscope (JEOL, Japan) at 80 kV. Images were processed using Adobe Photoshop software (Adobe Systems, Mountain View, CA).

### Cell viability assay

The cytotoxicity of the proteins was estimated by MTT assay as previously described [64]. The proteins in RPMI-1640 or X-VIVO 15 medium were added to the cell lines or hPBMCs, respectively. The media were supplemented with 10% FCS and 2 mM L-glutamine. Media for CTLL-2 and hPBMCs contained 100 units/ml IL-2. The incubation time for both CTLL-2 cells and hPBMCs with MTT was 2.5 h while other cell lines were incubated with MTT for 1 h. The A_540_ was measured with a Multiscan MCC/340 plate reader (Titertek, UK). The experiments were carried out in triplicate. Data (means±SD of triplicates) are expressed as the percentage of untreated controls.

### Preparation of SKOV-3 cells for the determination of mechanisms of cytotoxicity

Exponentially growing SKOV-3 cells were seeded at 30–40% confluence in 6-well plates. After an overnight incubation, the medium was changed and cells were cultured in the presence of either 50 µM barnase or 50 nM scFv 4D5-dibarnase for 24, 48, or 72 h (the time periods indicated in the figure legend). Both adherent and floating cells were harvested in PBS containing 5 mM EDTA, washed twice with cold PBS, and used for isolation of RNA, DNA, or for flow cytometry assays. SKOV-3 cells that were collected after 7 days of serum deprivation were used as positive controls for DNA fragmentation.

### RNA degradation assay

Aliquots of 2×10^5^ treated or mock-treated cells were processed for isolation of total RNA as described previously [65]. RNA pellets were dissolved in formamide buffer (95% formamide, 5 mM EDTA, 0.025% SDS, and 0.01% bromophenol blue) at 65°C for 10 min and the samples were loaded onto a 9% polyacrylamide gel containing 7.5 M urea. Gel images were analyzed by ImageJ software (Wayne Rasband National Institutes of Health). The percentage of degradation was calculated with respect to mock-treated controls. The alterations in the integral brightness of the corresponding RNA bands were analyzed.

### Measurement of DNA fragmentation by flow cytometry

After harvesting, cells were fixed in cold 70% ethanol and stored at −20°C. Prior to analysis, cells were washed with PBS and treated with 50 µg/ml RNase A in PBA for 30 min at 37°C. Cells were then stained with 10 µg/ml propidium iodide for 5 min and analyzed immediately with a FACScan flow cytometer (Becton Dickinson). For each determination, 10^4^ cells were counted. Cell cycle distribution percentages were calculated using Cell Quest software (Becton Dickinson). To calculate the alterations in the cell cycle distribution, the respective untreated controls were subtracted from the samples.

### Detection of apoptosis

Cell morphologies of treated and untreated cells were analyzed by phase contrast light microscopy using an inverted Axiovert 200 microscope. The DNA laddering assay and the incorporation of acridine orange into nuclear DNA were performed as described [56]. Apoptosis was quantified using the Vybrant Apoptosis Assay Kit #3 (Molecular Probes) according to the manufacturer's instructions. Caspase-3-like enzymatic activities were assessed by the cleavage of the fluorogenic substrate PhiPhiLux-G_1_D_2_ (OncoImmunin, Inc.) according to the manufacturer's instructions. Labeled cells were analyzed by flow cytometry as described above.
